# Insights into the Role of *GhTAT2* Genes in Tyrosine Metabolism and Drought Stress Tolerance in Cotton

**DOI:** 10.3390/ijms26031355

**Published:** 2025-02-05

**Authors:** Teame Gereziher Mehari, Jungfeng Tang, Haijing Gu, Hui Fang, Jinlei Han, Jie Zheng, Fang Liu, Kai Wang, Dengbing Yao, Baohua Wang

**Affiliations:** 1 School of Life Sciences, Nantong University, Nantong 226019, China; fiamieta21@gmail.com (T.G.M.); tangjunfeng60@gmail.com (J.T.); 15936135237@163.com (H.G.); fanghui8912@126.com (H.F.); jinleihan@ntu.edu.cn (J.H.); kwang5@ntu.edu.cn (K.W.); 2 State Key Laboratory of Cotton Bio-Breeding and Integrated Utilization, Institute of Cotton Research of Chinese Academy of Agricultural Sciences, Anyang 455000, China; zhengjieself@163.com (J.Z.); liufcri@163.com (F.L.)

**Keywords:** *Gossypium*, drought stress, tyrosine aminotransferase, tyrosine metabolism, RT-qPCR, VIGS

## Abstract

*Gossypium hirsutum* is a key fiber crop that is sensitive to environmental factors, particularly drought stress, which can reduce boll size, increase flower shedding, and impair photosynthesis. The aminotransferase (AT) gene is essential for abiotic stress tolerance. A total of 3 *Gossypium* species were analyzed via genome-wide analysis, and the results unveiled 103 genes in *G. hirsutum*, 47 in *G. arboreum*, and 53 in *G. raimondii*. Phylogenetic analysis, gene structure examination, motif analysis, subcellular localization prediction, and promoter analysis revealed that the *GhAT* genes can be classified into five main categories and play key roles in abiotic stress tolerance. Using RNA-seq expression and KEGG enrichment analysis of *GhTAT2*, a coexpression network was established, followed by RT-qPCR analysis to identify hub genes. The RT-qPCR results revealed that the genes *Gh_A13G1261*, *Gh_D13G1562*, *Gh_D10G1155*, *Gh_A10G1320*, and *Gh_D06G1003* were significantly upregulated in the leaf and root samples following drought stress treatment, with *Gh_A13G1261* identified as the hub gene. The *GhTAT2* genes were considerably enriched for tyrosine, cysteine, methionine, and phenylalanine metabolism and isoquinoline alkaloid, tyrosine, tryptophan, tropane, piperidine, and pyridine alkaloid biosynthesis. Under drought stress, KEGG enrichment analysis manifested significant upregulation of amino acids such as L-DOPA, L-alanine, L-serine, L-homoserine, L-methionine, and L-cysteine, whereas metabolites such as maleic acid, p-coumaric acid, quinic acid, vanillin, and hyoscyamine were significantly downregulated. Silencing the *GhTAT2* gene significantly affected the shoot and root fresh weights of the plants compared with those of the wild-type plants under drought conditions. RT-qPCR analysis revealed that *GhTAT2* expression in VIGS-treated seedlings was lower than that in both wild-type and positive control plants, indicating that silencing *GhTAT2* increases sensitivity to drought stress. In summary, this thorough analysis of the gene family lays the groundwork for a detailed study of the *GhTAT2* gene members, with a specific focus on their roles and contributions to drought stress tolerance.

## 1. Introduction

Climate change is a major cause of abiotic and biotic stresses that may adversely affect agricultural productivity to irreversible levels, thereby limiting production growth and jeopardizing sustainable agriculture globally [1]. It is a major cause of biotic and abiotic stresses that negatively affect global agricultural production and productivity [2]. It exacerbates drought conditions around the world, with significant implications for water availability and agricultural systems. It affects the frequency, severity and duration of droughts [3]. Droughts have significant impacts on agricultural production and food security, but global trends in how droughts affect agricultural production remain poorly understood. With more severe droughts expected due to climate change, assessing the vulnerability of agricultural production to these phenomena has become an important area of research [4]. This is the main reason for decreased crop yields caused by abiotic factors globally. This issue leads to food shortages and poses significant challenges for small-scale farmers, who struggle to grow enough grain during periods of low and unpredictable rainfall [5]. Drought stress affects crops differently depending on the growth stage, with vegetative stage stress reducing light interception and grain-filling stage stress causing leaf senescence [6]. This significantly impacts cotton production by reducing growth, photosynthesis, and yield [7].

Cotton is a globally significant crop that is cultivated on more than 30 million hectares, with production exceeding 70 million tons of seed cotton [8]. *Gossypium hirsutum*, commonly known as upland cotton, is an important fiber crop that is sensitive to various environmental factors. Drought stress is a major concern in cotton cultivation, leading to reduced boll size, increased flower shedding, and impaired photosynthesis. It also causes oxidative damage through the production of reactive oxygen species (ROS), which can be mitigated by natural defense enzymes [9]. Cotton responds to water deficit through various mechanisms, including stomatal closure, osmoregulation, and accumulation of plant growth regulators. At the cellular level, drought stress triggers the overproduction of reactive oxygen species (ROS) and activates signaling pathways involving mitogen-activated protein kinases, Ca^2+^, and hormones [10]. These responses lead to the expression of stress-related transcription factors and genes, particularly those involved in ROS scavenging and ABA signaling [11]. To mitigate the effects of drought stress, researchers have suggested strategies such as developing drought-tolerant cultivars, applying nutrients and osmoprotectants, and utilizing plant growth-promoting rhizobacteria [10]. Additionally, identifying drought tolerance traits through QTL analysis and transgenic approaches may increase cotton resilience to water scarcity [11].

Aminotransferase genes are vital for improving stress tolerance in crops through the regulation of proline synthesis [12]. Classes of the aminotransferase family are involved in the metabolism of various amino acids, including aromatic amino acid aminotransferases (AAA-ATs) [13], tyrosine aminotransferase (*TAT*) [14], alanine aminotransferase (ALT) [15], aspartate aminotransferase (AST) [16], and histidinol-phosphate aminotransferase (HisC) [17]. Tyrosine aminotransferase (*TAT*) is essential for the growth and development of plants. The methyl jasmonate (MeJA)-induced *TAT* gene reacts to a variety of abiotic stressors. The *TAT* activity of poplar roots is increased by drought and low nitrogen stress, which alters the amount of RA [18]. TATs are essential for tyrosine metabolism and degradation, with *TAT1* and *TAT2* exhibiting both distinct and overlapping functions in *A. thaliana*. The majority of current research indicates that At*TAT1* aids in plant survival in the dark and is involved in tyrosine catabolism [19]. The first enzyme involved in the metabolic breakdown of tyrosine is tyrosine aminotransferase (*TATN*), which is crucial for tyrosine detoxification and assisting the body in fending off oxidative damage [20]. The *TAT* gene plays a crucial role in the synthesis of tocopherols in Arabidopsis. The study revealed that knockout mutants presented a substantial reduction in total *TAT* activity, leading to a significant accumulation of tyrosine and a marked decrease in tocopherol levels. These findings indicate that *TAT* is important for the utilization of tyrosine in metabolic pathways that produce tocopherols, which are vital for plant health and have significant nutritional value [21].

Multiple studies have highlighted the role of *TAT* genes across various crop species. *SmTAT3-2* is involved in phenolic acid biosynthesis in *Salvia miltiorrhiza* [22], whereas the overexpression of *MdTAT2* improves drought and osmotic stress tolerance in *Malus domestica* [13]. In Arabidopsis, *AtTAT1* and *AtTAT2* are linked to tyrosine metabolism and degradation, supporting plant survival [19,23]. Additionally, the *AccTATN* gene helps *Apis cerana cerana* respond to pesticide and heavy metal stress [20]. In this study, the *GhTAT2* gene from *G. hirsutum* was cloned for gene knockdown, and the expression patterns of this gene family were analyzed. Recombinant *GhTAT2* proteins were subjected to drought stress tests in cotton seedlings, and their role in tyrosine metabolism and other metabolic pathways was examined. This is the first functional characterization of *TAT2* in *G. hirsutum* related to stress tolerance. These findings provide valuable insights into the role of *TAT2* genes in the stress response and tyrosine metabolic pathways in cotton.

## 2. Results

### 2.1. Genome-Wide Identification of Aminotransferase Genes from Cotton Species

The 3 cotton species included a total of 203 genes, with 103 genes in *G. hirsutum* (47 in the GhAt subgenome and 53 in the GhDt subgenome) and 47 and 53 genes in the diploid species *G. arboreum* and *G. raimondii*, respectively. The A genome species (*G. arboreum*) and D genome species (*G. raimondii*) are regarded as progenitors of cultivated *G. hirsutum* [24]. The lengths of the *TAT2* gene proteins ranged from 1068 to 3261 bp in the GhAt subgenome of *G. hirsutum*, 333 to 3378 bp in the GhDt subgenome, 537 to 3360 bp in *G. arboreum*, and 570 to 4083 bp in *G. raimondii*. The protein lengths ranged from 355 to 1086 aa in the *G. hirsutum* GhAt subgenome, 53 to 1125 aa in the GhDt subgenome, 178 to 1119 aa in *G. arboreum*, and 170 to 1084 aa in *G. raimondii*. The molecular weights varied from 38.734 to 121.045 kDa in the GhAt subgenome, 5.688 to 123.409 kDa in the GhDt subgenome, 20.782 to 122.993 kDa in *G. arboreum*, and 18.705 to 121.023 kDa in *G. raimondii*. The isoelectric points ranged from 5.03 to 9.495 in the GhAt subgenome, 4.324 to 10.748 in the GhDt subgenome, 5.418 to 9.575 in *G. arboreum*, and 4.826 to 10.43 in *G. raimondii* (Supplementary File S1).

### 2.2. Phylogenetic Tree Classification of Aminotransferase Proteins

**Figure 1:**
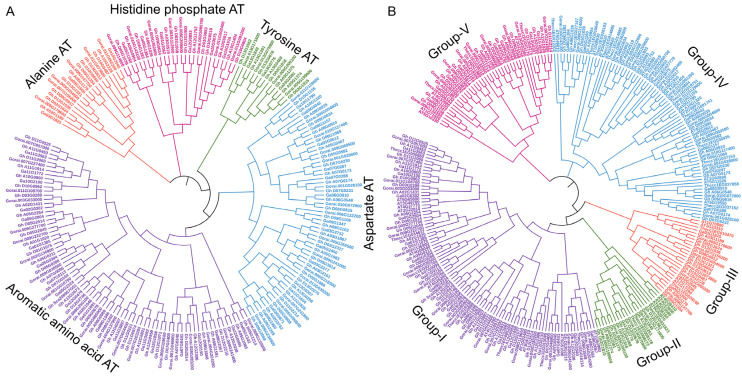
Phylogenetic tree analysis of aminotransferase gene family members. (**A**) Tree constructed using the protein sequences of *G. hirsutum*, *G. arboreum* and *G. raimondii*. (**B**) Tree constructed using the protein sequences of *G. hirsutum*, *G. arboreum*, *G. raimondii*, *A. thaliana* and *T. cacao*. Different colors represent different clusters, and AT stands for aminotransferase.

This study systematically searched for aminotransferase genes in the genomes of diploid and tetraploid cotton, Arabidopsis, and tobacco via BLAST and identified 103, 47, 53, 28, and 34 aminotransferase genes in *G. hirsutum*, *G. arboreum*, *G. raimondii*, *T. cacao*, and *A. thaliana*, respectively. This study focused primarily on tetraploid *G. hirsutum* to explore the origin and evolution of polyploidy by analyzing aminotransferase genes. Additionally, comparisons with two other cotton species, *A. thaliana* and *T. cacao*, which are close relatives of upland cotton, were performed to elucidate their evolutionary history. To trace the evolutionary history of aminotransferase genes, we constructed a phylogenetic tree via protein sequences from multiple alignments of 103 *G. hirsutum*, 47 *G. arboreum*, 53 *G. raimondii*, 28 *T. cacao*, and 34 *A. thaliana* crop species. The alignment was performed with CLUSTALX (version 2.0) software, and an unrooted phylogenetic tree was generated via maximum likelihood (ML) via the online program iTOL (version 6) [25]. Among the 3 *Gossypium* species, the aminotransferase tree formed 5 clusters: 80 proteins encoding aromatic amino acid aminotransferases, 15 encoding tyrosine aminotransferases, 20 encoding alanine aminotransferases, 60 encoding aspartate aminotransferases, and 28 encoding histidine phosphate aminotransferases (Figure 1A). Similarly, of the 5 species, including *A. thaliana* and *T. cacao*, 94 proteins clustered in group I, 23 in group II, 26 in group III, 79 in group IV, and 43 in group V, each represented with different colors (Figure 1B).

### 2.3. Chromosomal Location, Gene Architecture and Conserved Domain Distribution

**Figure 2:**
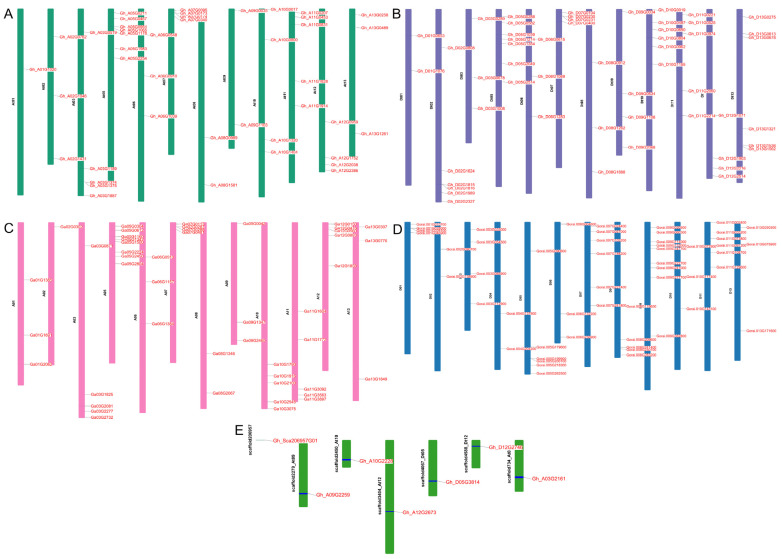
Chromosome mapping of aminotransferase genes. (**A**) *G. hirsutum* GhAt subgenome; (**B**) *G. hirsutum* GhDt subgenome; (**C**) *G. arboreum*; (**D**) *G. raimondii*; (**E**) genes located in scaffold regions.

Chromosomal mapping of AT genes from *G. hirsutum* (AD)_1_ and two diploid cotton species, *G. arboreum* (AA) and *G. raimondii* (DD), was performed to investigate their structural and evolutionary dynamics related to genomic distribution. Among the 103 *G. hirsutum genes*, 43 were found on 12 chromosomes of the At subgenome (GhAt), with chromosomes 05 and 01 having the largest and lowest numbers of genes, with 7 and 1 genes, respectively. A total of 53 aminotransferase genes were irregularly distributed across 12 chromosomes in GhDt. The highest and lowest number of genes, 07 and 02, are found on chromosomes 05 and 01, respectively. Neither the GhAt nor the GhDt subgenomes contained any genes on chromosome number four. In both subgenomes, four GhAt genes and three GhDt genes were identified as scaffolds (Figure 2A,B,E). Similarly, in *G. arboreum*, 47 aminotransferase genes are dispersed at random among the A-genome’s 12 chromosomes, excluding chromosome 04. The greatest number of aminotransferase genes are found on chromosome A05 (8), and the fewest are found on chromosome A02 (1). Except for chromosome D12, all 12 chromosomes of the D-genome in *G. raimondii* contain 53 aminotransferase genes. D09 has the most genes (9), whereas D02 and D04 have the fewest (2 genes). Neither of the diploid species had scaffolds (Figure 2C,D).

A total of 203 aminotransferase genes were classified into 3 subgroups according to their exon and intron counts. The intron-exon structure of aminotransferase genes was visualized to assess the evolutionary impact on the conservation or divergence of introns and exon numbers in cotton. The results revealed that exon counts ranged from 2 to 15 in the GhAt subgenome of *G. hirsutum*, 2 to 26 in the GhDt subgenome of *G. hirsutum*, 2 to 25 in *G. arboreum*, and 3 to 15 in the aminotransferase gene family of *G. raimondii* (Figure S1) The arrangement of exons and introns illuminates the evolutionary relationships among different gene family members. Notably, closely related genes within the same phylogenetic clade presented greater structural similarity in terms of intron and exon numbers than did those in other clades. A positive correlation was observed between phylogeny and exon-intron structure. Additionally, a one-intron-less structure was identified in the scaffold region of *Gh_Sca206957G01*. The absence of introns in these *aminotransferase* genes suggests a lower likelihood of alternative splicing.

**Figure 3:**
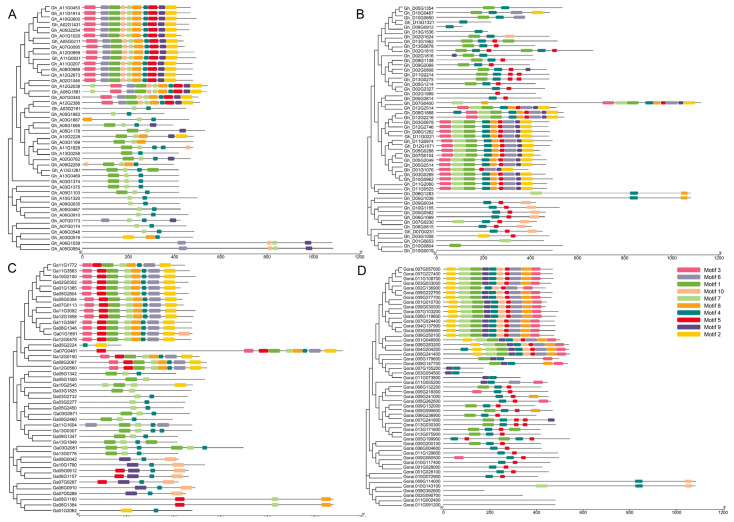
Phylogenetics, gene structure and motif analysis of the aminotransferase genes in cotton species: (**A**) the *G. hirsutum* GhAt subgenome (**B**), the *G. hirsutum* GhDt subgenome, (**C**) *G. arboreum* and (**D**) *G. raimondii*. The phylogenetic tree was created with MEGA 7 via the neighbor-joining method and 1000 bootstrap replicates, while conserved motifs in aminotransferase proteins were visualized with TBtools (version 2.154) software. The exon-intron structures of the aminotransferase genes reflect their evolutionary relationships, with yellow circles representing exons and gray lines indicating introns. Each motif is marked by a colored box in the legend, and the lengths of the motifs in each protein are shown proportionally.

Moreover, all cotton aminotransferase protein sequences were analyzed via MEME to identify coding motifs, revealing that aminotransferase proteins contain between one and ten conserved motifs. Among them, in the GhAt subgenome, motifs 4 and 7; in the GhDt subgenome, motifs 1, 4 and 5; in *G. arboreum*, motifs 1, 4 and 7; and in *G. raimondii*, motifs 4 and 5 remained almost conserved in all the proteins across the three species of cotton (Figure 3A–D). Specifically, we identified genes with no motif in the *Gh_D10G0019* gene of the GhDt subgenome and the *Gorai.009G362800*, *Gorai.002G090700*, *Gorai.011G002400* and *Gorai.011G091200* genes in the *G. raimondii* genome (Figure 3D). Furthermore, all aminotransferase genes presented a conserved protein motif distribution pattern. For example, motif number 4 was observed in almost all aminotransferase proteins. Overall, aminotransferase genes show a strong ancestral link in their gene structure and phylogeny, maintaining consistent patterns of gene structure and protein motif distribution across subfamilies.

### 2.4. Analysis of Cis Regulatory Elements and Their Subcellular Localization

**Figure 4:**
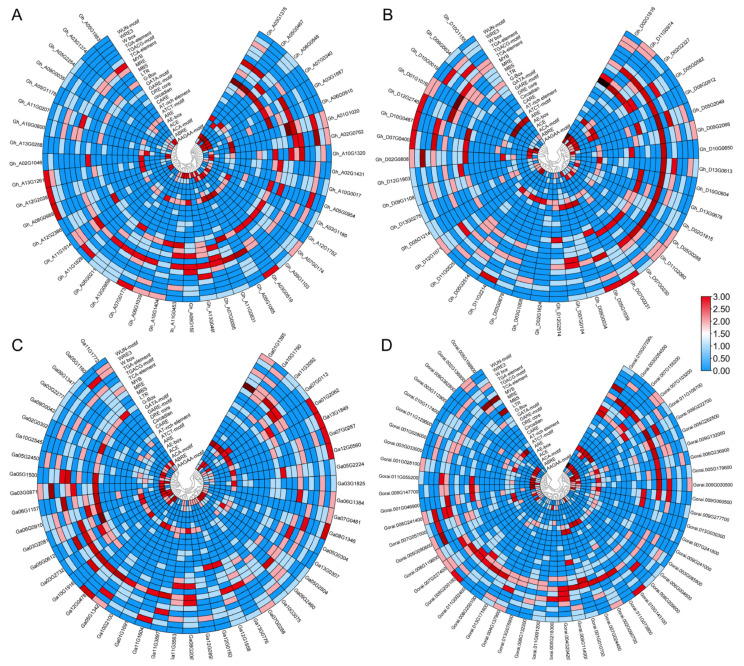
*Cis*-regulatory element analysis of the promoter regions of the aminotransferase gene family. (**A**) *G. hirsutum* GhAt subgenome; (**B**) *G. hirsutum* GhDt subgenome; (**C**) *G. arboreum*; (**D**) *G. raimondii*.

Analysis of the *cis*-acting regulatory elements in the promoter regions revealed that aminotransferase genes play various roles. ABRE, ARE, MBS, and MYB elements are associated with abscisic acid responsiveness, drought inducibility, and anaerobic induction. Elements such as the G-Box, GATA motif, LTR, and MRE are associated with light and low-temperature responsiveness. Similarly, motifs such as GARE, TGACG, TCA, and TGA are involved in hormone signaling related to auxin, gibberellin, MeJA, and salicylic acid (Figure 4A–D).

**Figure 5:**
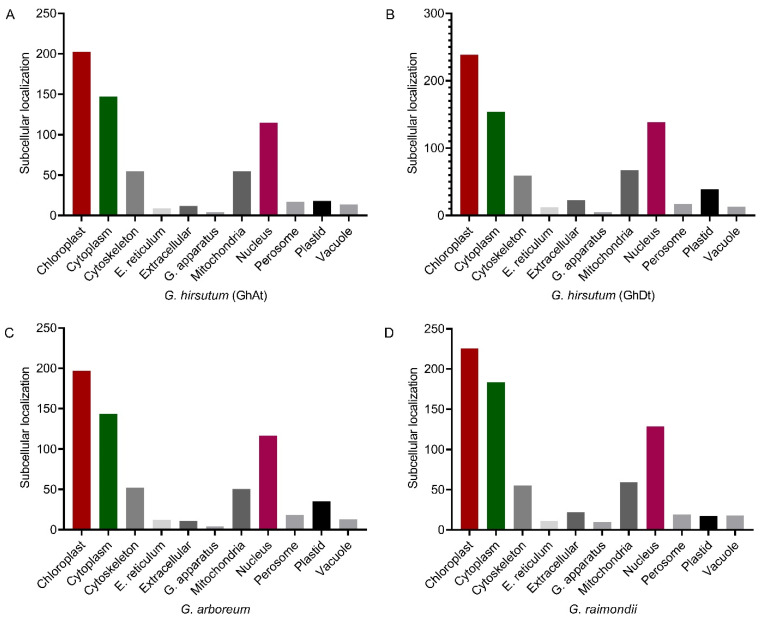
Prediction of the subcellular localization of aminotransferase genes in *Gossypium* species. (**A**) *G. hirsutum* in the GhAt subgenome; (**B**) *G. hirsutum* in the GhDt subgenome; (**C**) *G. arboreum*; (**D**) *G. raimondii*.

According to other prediction analyses, the three *Gossypium* species are expected to be present in various cellular compartments, including the chloroplast, cytoplasm, cytoskeleton, endoplasmic reticulum, extracellular space, Golgi apparatus, mitochondria, nucleus, peroxisome, plastids, and vacuole. Most genes are located predominantly in the chloroplast, cytoplasm, and nucleus, with fewer genes scattered in the other compartments (Figure 5A–D). The results indicated that most aminotransferase gene family members are highly expressed in the chloroplast, cytoplasm, and nucleus of plant cells, suggesting their significant role in food synthesis through photosynthesis, tolerance against stress, and regulation of cell functions.

### 2.5. Coexpression Network Analysis of Aminotransferases in G. hirsutum

**Figure 6:**
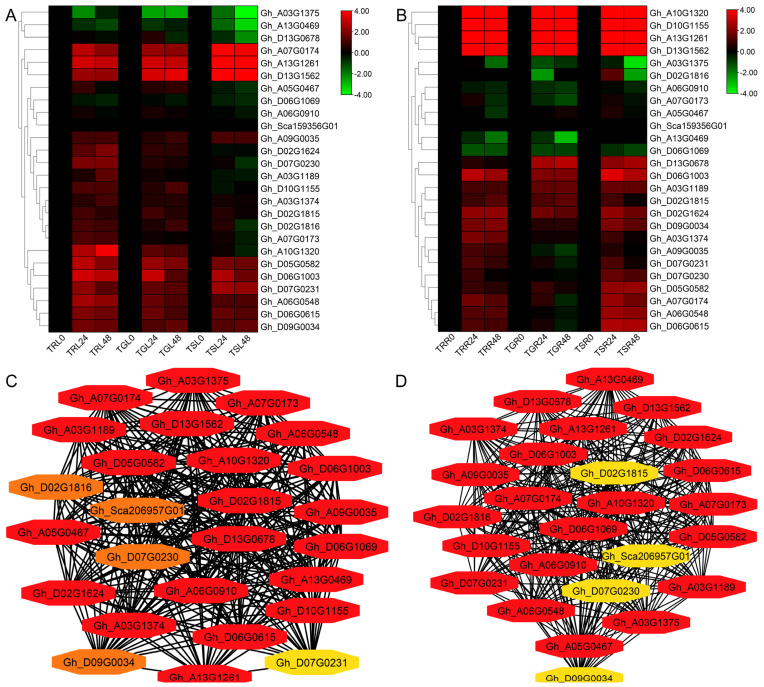
Transcriptome and coexpression network analysis of aminotransferase genes. (**A**) Transcriptome analysis of aminotransferase genes in *G. hirsutum* leaf tissue; (**B**) transcriptome analysis of aminotransferase genes in *G. hirsutum* root tissue; (**C**) coexpression analysis and candidate gene identification in leaf expression; (**D**) coexpression analysis and candidate gene identification in root expression. Genes in red have positive correlation values and are considered hub genes.

A total of 25 genes from the aminotransferase gene family involved in various metabolic pathways were used to analyze gene coexpression networks via RNA-seq data from the leaf and root tissues of *G. hirsutum* (Figure 6A,B). The Cytohubba program in Cytoscape software (version 3.10.2) was used to analyze the regulatory network of *aminotransferase* gene coexpression, with a *p* value of 0.95 used as the threshold. Key genes were selected from leaf and root tissues on the basis of the range of topological coefficients associated with each node. *Gh_A13G1261*, *Gh_D13G1562* and *Gh_D10G1155* were identified as hub genes from both the leaf and root tissue network analyses (Figure 6C,D).

### 2.6. Identification of Drought-Responsive Candidate Genes

**Figure 7:**
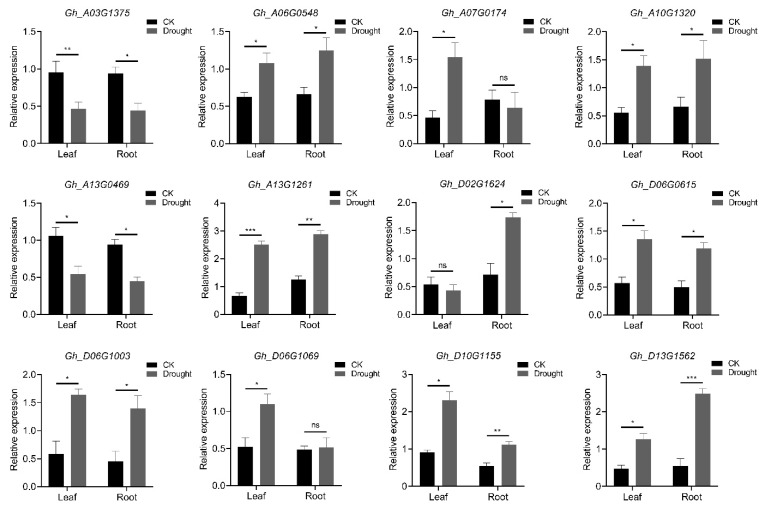
The relative expression levels of aminotransferase genes after drought stress were analyzed via a *t*-test, and the results are presented as the means ± SDs from three independent experiments. Significance is indicated by *, **, and *** for *p* ≤ 0.05, *p* ≤ 0.01, and *p* ≤ 0.001, respectively, ns stands for non-significant.

We selected 12 DEGs from the aminotransferase family involved in metabolite pathway enrichment for real-time quantitative validation. After drought stress treatment, *Gh_A13G1261*, *Gh_D13G1562*, *Gh_D10G1155*, *Gh_A10G1320*, and *Gh_D06G1003* were significantly upregulated in both materials, whereas *Gh_A03G1375* and *Gh_A13G0469* were significantly downregulated in both tissues. The expression of *Gh_A07G0174* and *Gh_D06G1069* did not significantly change in root tissue, whereas that of *Gh_D02G1624* did significantly change in leaf tissue after drought stress. *Gh_A13G1261*, a member of the tyrosine aminotransferase class (*GhTAT2*), was chosen as the candidate gene for functional characterization (Figure 7). These results indicate that drought stress induces unique gene expression trends in cotton seedlings with different drought tolerances, influencing various pathways involved in the drought response and accounting for the observed tolerance variations. The RT-qPCR results for these genes aligned with the transcriptome expression trends. This shows a strong correlation between the transcriptome sequencing and RT-qPCR results, confirming the reliability and effectiveness of the data. In conclusion, the *AT* gene can rapidly adjust its expression through transcription within 48 h of drought stress to increase drought tolerance in cotton.

### 2.7. Metabolite Pathway Enrichment Analysis

**Figure 8:**
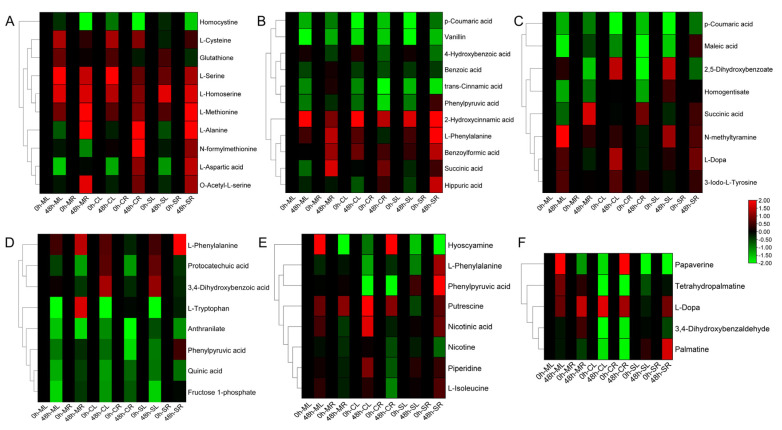
Metabolite enrichment analyses of *GhTAT2* genes under drought stress. (**A**) Cysteine and methionine metabolism; (**B**) phenylalanine metabolism; (**C**) tyrosine metabolism; (**D**) phenylalanine, tyrosine and tryptophan biosynthesis; (**E**) tropane, piperidine and pyridine alkaloid biosynthesis; (**F**) isoquinoline alkaloid biosynthesis. The heatmap was generated in TBtools via log2-transformed metabolite expression data.

KEGG enrichment analysis was conducted on *G. hirsutum* races to identify key and significantly enriched metabolites that are vital for drought stress tolerance. The *GhTAT2* gene is involved in and regulates various metabolic pathways, namely, tyrosine metabolism; cysteine and methionine metabolism; isoquinoline alkaloid biosynthesis; phenylalanine metabolism; phenylalanine, tyrosine and tryptophan biosynthesis; and tropane, piperidine and pyridine alkaloid biosynthesis (Figure 8A–F). In cysteine and methionine metabolism, L-alanine, L-serine, L-homoserine, and L-methionine amino acids were upregulated, whereas homocystine was downregulated (Figure 8A). In tyrosine metabolism, phenylalanine, tyrosine and tryptophan biosynthesis amino acids, such as L-phenylalanine, L-DOPA, N-methyltyramine and succinic acid, were significantly upregulated, whereas maleic acid, p-coumaric acid, quinic acid, fructose 1-phosphate, phenylpyruvic acid and anthranilate were significantly downregulated (Figure 8C,D). In phenylalanine metabolism, isoquinoline alkaloid biosynthesis and tropane, piperidine and pyridine alkaloid biosynthesis metabolites such as 2-hydroxycinnamic acid, L-phenylalanine, benzoic acid, putrescine and L-DOPA were significantly upregulated, whereas papaverine, p-coumaric acid, vanillin, hyoscyamine and nicotine were downregulated after drought stress treatment (Figure 8B,F). A list of the metabolite data used for the enrichment analysis is provided in Supplementary File S2.

### 2.8. VIGS Agroinfiltration, Drought Stress Treatment and Relative Expression Analysis

**Figure 9:**
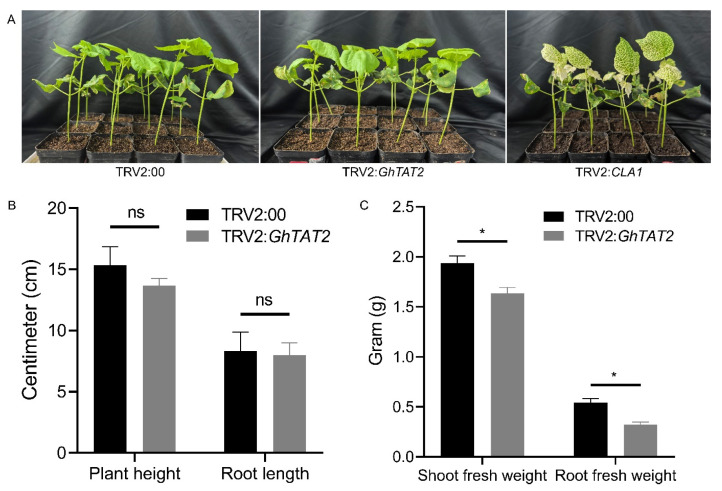
Phenotypic variations in cotton seedlings after agroinfiltration (**A**) TRV2:00, empty vector, TRV2:*GhTAT2*, VIGS plant, TRV2:*CLA1*, positive control, (**B**) plant height and root length measurement, and (**C**) shoot fresh weight and root fresh weight measurement. ns stands for non-significant; * indicates a significant difference at *p* < 0.05.

To assess the role of the *GhTAT2* gene (*Gh_A13G1261*) in drought stress tolerance, *GhTAT2* gene silencing was performed on cotton seedlings. The experimental setup included TRV2:00, TRV2:*GhTAT2*, and TRV2:*CLA1*, with TRV2:*CLA1* exhibiting an albino phenotype in leaf tissues from the second week onward (Figure 9A). Significant differences (*p* < 0.05) in morphological traits were noted among VIGS-treated plants and positive controls under drought conditions. Although plant height and root length remained similar, the VIGS-treated plants presented significantly lower shoot and root fresh weights than the positive controls did (Figure 9B,C). Samples were collected from the leaves and roots of TRV2:00 and TRV2:*GhTAT2* plants to investigate the role of the *GhTAT2* gene under drought conditions.

## 3. Discussion

Cotton (*Gossypium hirsutum* L.) is a commercially valuable fiber crop grown worldwide in various climates, with increasing demand driven by its use in the textile and oil industries [26]. This versatile crop is vulnerable to biotic and abiotic stresses, especially high temperatures and drought, which can considerably affect yield and quality [27]. Drought stress is a pressing global issue that significantly impacts cotton production. There is an increasing need to identify or breed drought-tolerant varieties to ensure sustainable cotton farming. This stress hinders cotton growth and development by altering metabolic pathways, lowering photosynthesis, and causing responses such as stomatal closure. It decreases photosynthesis and reduces the supply of photosynthates, which results in boll shedding and a low lint yield [10,28]. It impacts several physiological processes in plants, including photosynthesis, stomatal regulation, root–shoot growth ratio, leaf area expansion, transpiration, and osmoregulation. This stress triggers sensing and signaling pathways that activate various parallel responses, including physiological, molecular, and biochemical mechanisms [29,30]. Drought stress at the cellular level triggers excessive production of reactive oxygen species, mitogen-activated protein kinases, Ca^2+^, and hormone-mediated signaling. It also activates transcription factors involved in both abscisic acid-dependent and abscisic acid-independent stress signaling pathways in cotton. Cotton plants adapt to drought through mechanisms such as the accumulation of reactive oxygen species (ROS) and the activation of stress-responsive transcription factors [7,10].

This study identified 103, 47, 53, 28, and 34 aminotransferase genes in *G. hirsutum*, *G. arboreum*, *G. raimondii*, *A. thaliana*, and *T. cacao,* respectively. The number of genes in cottons were greater than Arabidopsis, and tobacco. This is because of the polyploidy nature of cotton species due to interspecific hybridization. There are diploid and tetraploid sets of chromosomes in cotton, while Arabidopsis has only two sets. This extra genetic evolution in cotton gives it more genes for various functions [31]. The phylogenetic analysis grouped the aminotransferase class into 5 different classes, with 94, 23, 26, 79 and 43 aminotransferase proteins in the I, II, III, IV, and V groups, respectively. In the *Jinjiang oyster*, 18 aminotransferase class I and II genes were identified, and their expression varied under salinity stress [32]. Aminotransferases are categorized into five classes: I, II, III, IV, and V. Classes I and II include alanine aminotransferase, aromatic amino acid aminotransferase, aspartate aminotransferase, and histidine phosphate aminotransferase [32,33]. Aminotransferases are essential enzymes responsible for amino acid metabolism and transfer [33]. The aminotransferase gene family plays crucial roles in amino acid metabolism and osmotic regulation across various organisms [32]. Across species, aminotransferases are involved in crucial cellular processes, including osmotic pressure regulation, nitrogen metabolism, and stress response, highlighting their importance in cellular adaptation and homeostasis [32,34].

In the subcellular localization prediction analysis, most genes were found to be located in the chloroplast, cytoplasm, or nucleus. These cell components play crucial roles in photosynthesis, regulating cell functions, and enhancing stress tolerance. During stress, chloroplasts synthesize essential compounds such as amino acids, vitamins, phytohormones, lipids, nucleotides, and secondary metabolites [35]. Similarly, cytoplasmic stress granules and cytosolic pH homeostasis act as signaling hubs that influence cell viability and stress recovery and are essential for normal growth and stress responses in plants [36,37]. In addition, nucleolar proteins are essential for stress adaptation, affecting plant growth and tolerance to environmental stresses [38]. Analysis of *cis*-acting elements in the promoter regions of *aminotransferase* genes revealed several important motifs, including ABREs, AREs, MBSs, MYBs, G-boxes, GATA, LTRs, MREs, GAREs, TGACGs, TCAs, and TGATAs, which are associated with stress tolerance, light regulation, and hormone signaling. Various studies have identified motifs involved in hormonal regulation and phytohormonal responses, including ABREs, GARE motifs, GATA transcription factors [39,40,41], G-box, GT1 motifs, TCT motifs, MREs, and GATA motifs, which regulate development and responses to abiotic stresses [42]. These findings indicate that *TAT2* is important for plant growth and abiotic stress resistance.

The *aminotransferase* gene plays a crucial role in abiotic stress tolerance, as revealed by KEGG enrichment analysis of pathways such as tyrosine metabolism, cysteine and methionine metabolism, and phenylalanine metabolism. Key metabolites, including N-methyltyramine, succinic acid, L-alanine, L-serine, L-homoserine, L-cysteine, L-methionine, 2-hydroxycinnamic acid, benzoic acid, and L-phenylalanine, were upregulated, indicating that aminotransferases are involved in drought stress tolerance. Tyrosine aminotransferases play a role in the oxidative stress response through m-tyrosine metabolism in *Caenorhabditis elegans* [43]. In *Vigna radiata*, tyrosine and lysine enhance growth under cadmium stress, highlighting their importance in plant nitrogen metabolism [44]. Additionally, tyrosine metabolism may improve drought tolerance by affecting carbon and nitrogen metabolism in okra [45]. The tyrosine metabolism pathway initiates the production of various structurally diverse natural compounds in plants, including tocopherols, betalains, salidroside, plastoquinone, ubiquinone, and benzylisoquinoline alkaloids. Notably, tyrosine-derived metabolites, tocopherols, ubiquinone and plastoquinone are vital for plant survival [46].

Plants generate several l-tyrosine (Tyr)-derived compounds essential for their adaptation, as well as having pharmaceutical and nutritional significance for human health. *TAT* catalyzes the reversible reaction between Tyr and 4-hydroxyphenylpyruvate, serving as the entry point for the biosynthesis of various natural products and the degradation of Tyr for energy and nutrient recycling [19]. Similar *TAT* genes have been found in other plants, including *Salvia miltiorrhiza*. In this species, three *SmTAT* genes exhibit various expression patterns and react to methyl jasmonate stimuli [47]. Methionine is crucial in the oxidative stress response and functions as a scavenger of reactive oxygen species [48]. It is central to the interconversion of sulfur-containing amino acids, with cysteine contributing to oxygen tolerance [49]. Both methionine and cysteine are highly sensitive to various reactive oxygen species, highlighting their antioxidant properties [50]. Furthermore, phenylalanine metabolism plays a crucial role in mitigating the negative effects of drought in *Brassica campestris* [51], cold stress in tartary buckwheat landraces [35], and heat stress in *Cucumis sativus* [52]. The enhanced production of aromatic amino acids in tobacco plants results in a significant increase in the levels of phenylpropanoid metabolites. This, in turn, contributes to improved tolerance against stresses that the plants may encounter [53]. Similarly, TAT enzymes are involved in various metabolic pathways, like amino acid metabolism, vitamin biosynthesis and secondary metabolites [33].

**Figure 10:**
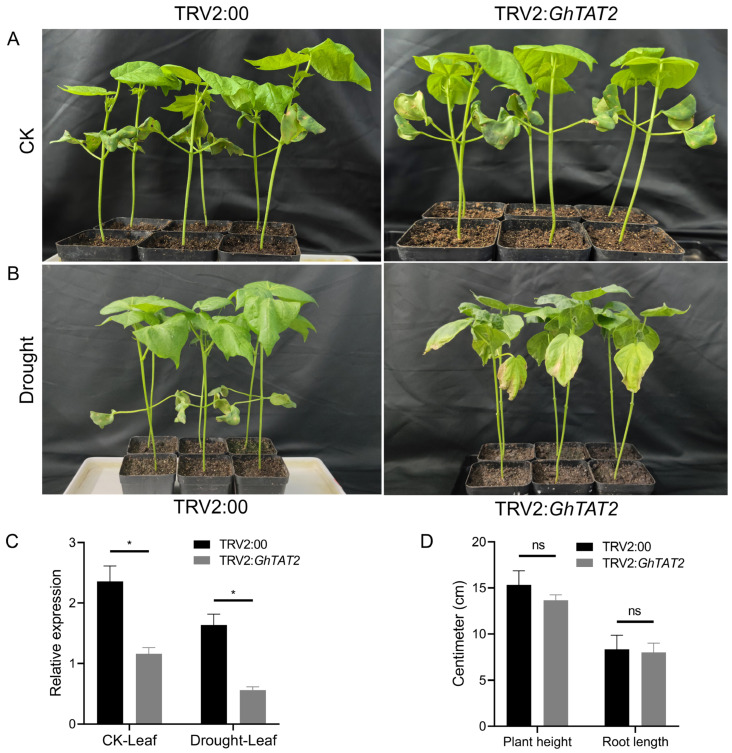
Relative expression analysis of positive control and silenced tissues. (**A**) Phenotypic images of TRV2:00 and TRV2:*GhTAT2* before treatment. (**B**) Phenotypic images of TRV2:00 and TRV2:Gh*TAT2* after PEG-6000 treatment. (**C**) Expression profiling of the empty vector and TRV2:*GhTAT2* before drought stress treatment in leaf and root tissues. (**D**) Expression profiling of the empty vector and TRV2:*GhTAT2* after drought stress treatment in leaf and root tissues. Bars with different letters indicate significant differences at * for *p* ≤ 0.05, ns stands for non-significant.

VIGS analyses revealed phenotype differences in TRV2:00 and TRV2:*GhTAT2* before and after drought stress treatment, highlighting its potential role in cotton drought tolerance (Figure 10A,B). RT-qPCR analysis disclosed that *GhTAT2* expression in VIGS-treated plants was lower than that in positive control plants, indicating that TRV2:*GhTAT2* is sensitive to drought stress. Additionally, the morphological traits of VIGS-treated seedlings were notably different from those of TRV:00-treated seedlings. Silencing the *GhTAT2* gene reduced both shoot and root fresh weight. Furthermore, after drought, the relative expression of the *GhTAT2* gene significantly increased in the leaves and roots of positive control plants but notably decreased in both tissues of VIGS-treated plants (Figure 10C,D). Previous reports indicate that the *TAT2* gene enhances stress responses in various crop species. In *Populus simonii*, *TAT2* genes improve drought tolerance and low nitrogen tolerance [18]. The overexpression of *MdTAT2* in *Malus domestica* enhances resistance to osmotic and drought stress [13], whereas the overexpression of *AccTATN* in *Apis cerana cerana* increases heavy metal stress tolerance and antioxidant capacity [20]. An ornithine δ-aminotransferase gene *OsOAT* gene, plays a crucial role in the metabolism of proline and arginine. This gene has been found to enhance drought tolerance as well as provide protection against oxidative stress in rice plants [54].

## 4. Materials and Methods

### 4.1. Aminotransferase Gene Identification in Cotton

Cotton species genome data for *G. hirsutum* (NAU assembly), *G. arboreum* (CRI assembly) and G*. raimondii* (JGI assembly) were retrieved from CottonFGD (https://cottonfgd.org/, accessed on 12 December 2024) [55]. The protein sequences of *Theobroma cacao* (Ver 1.1) and *Arabidopsis thaliana* (TAIR 10) were obtained from the Phytozome database (https://phytozome-next.jgi.doe.gov/, accessed on 12 December 2024) [56]. To identify aminotransferase proteins, Pfam (https://pfam.xfam.org/, accessed on 18 December 2024) and HMMER (version 3.4) software were used with default settings to obtain aminotransferases containing the Pfam PF00155 domain, which probably belongs to the aminotransferase gene family. Incomplete and repeated sequences with an e value greater than 1 × 10^−5^ were removed [57]. We analyzed various gene features, protein statistics, and transcript characteristics of cotton aminotransferase genes, including lengths of CDSs, proteins and genes, molecular weights (MWs), isoelectric points (PIs), exon/intron lengths, and start and end codons, via CottonFGD [55].

### 4.2. Protein Sequence Alignment and Phylogenetic Tree Construction

The ClustalX tool (Ver. 2.1) was used to fully align all of the aminotransferase protein sequences identified from the five species and perform bootstrap N-J tree analysis with the default settings [58]. A Newick file was produced after manual modification in MEGA 7.0 software via the maximum likelihood approach with 1000 bootstrap repetitions [59]. The Newick file from MEGA 7.0 was used to construct the final tree via the iTOL online tool [25]. Comparative phylogenetic analysis was also performed to identify the evolutionary relationships of cotton aminotransferase with *A. thaliana* and *T. cacao*. All relevant list of proteins associated with aminotransferases are given in Supplementary File S3.

### 4.3. Chromosomal Mapping, Gene Structure and Conserved Domain Analysis

General feature format (GFF3) files and genome assembly sequences for the three cotton species were obtained from CottonFGD [55]. TBtools software [60] was used to visualize the chromosomal locations of aminotransferase genes in the cotton genomes. Gene structure analysis involved the retrieval of detailed coding sequence (CDS) and genomic sequences from CottonFGD. The intron/exon arrangement of the cotton aminotransferase genes was illustrated via the gene structure display server GSDS 2.0 (https://gsds.gao-lab.org/, accessed on 21 December 2024) [61]. Similarly, using protein sequences as inputs on the MEME Suite 5.5.7 online server (https://meme-suite.org/meme/, accessed on 21 December 2024), we identified the highly conserved motifs of aminotransferase proteins [62].

### 4.4. Promoter Region Analysis and Prediction of Subcellular Localization

We utilized the PlantCARE (https://bioinformatics.psb.ugent.be/webtools/plantcare/html/, accessed on 21 December 2024) webserver to identify cis-acting regulatory elements in the promoter regions of aminotransferase genes in *Gossypium* species [63]. The 2000 bp promoter sequences of aminotransferases from the upstream region were sourced from the CottonFGD database [55]. Protein sequences were used to determine the subcellular localization of aminotransferase proteins from *Gossypium* species via the WoLF PSORT online prediction tool (https://wolfpsort.hgc.jp/, accessed on 21 December 2024) [64].

### 4.5. Expression Profiling, Coexpression Network and RT-qPCR Analysis

RNA-seq data (NCBI accession number: PRJNA663204) were used to examine the relative expression patterns of aminotransferases under drought stress for 0 h, 24 h and 48 h for each treatment and two tissues, namely, roots and leaves, from three cotton races [65]. To verify the sequencing results, the identified candidate genes were analyzed via real-time quantitative PCR. The primers for the target genes (Supplementary File S4) were designed via NCBI Primer Blast (https://www.ncbi.nlm.nih.gov/tools/primer-blast/, accessed on 12 December 2024). Cytoscape software (version 3.10.2) was utilized for coexpression network analysis of leaf and root tissue FPKM values from RNA-seq data. The top 25 genes associated with various metabolic pathways were imported into Cytoscape based on their correlation values. The FPKM values of leaf and root tissues at 0 h, 24 h, and 48 h under drought stress were used to determine the correlations between *G. hirsutum* genes via a correlation calculation tool with default settings [66]. RT-qPCR was performed with the ChamQ SYBR qPCR Master Mix (LowROX Premixed) kit on a SLAN-96S real-time PCR system (Hongshi Medical Technology, Shanghai, China). The 20 μL RT-qPCR mixture comprised 10 μL of ChamQ SYBR qPCR Master Mix (Vazyme Biotech, Nanjing, China), 0.4 μL of each forward and reverse primer, 2 μL of cDNA template, and 7.2 μL of dd water. *GhUBQ* served as the internal reference, and the results were analyzed via the 2^−ΔΔCT^ method [67]. A heatmap was generated via TBtools software [60] with FPKM values for relative expression analysis, incorporating three biological and technical replicates.

### 4.6. Cotton Seedlings and Growth Conditions

The experiment took place in seedling growth chamber at Nantong University’s School of Life Sciences. For VIGS experiment, CRI variety, a *G. hirsutum* drought-tolerant cotton variety released by the Institute of cotton research, Chinese Academy of Agricultural Sciences (ICR, CAAS) was used [68]. Seedlings were planted in pots containing equal parts vermiculite and humus and were grown under the same conditions. A total of 12 pot cotton seedlings for the TRV2:00, TRV2:*GhTAT2* and TRV2:*CLA1* treatment sets were used for the experiment. When the seedlings grew to three true leaves, drought stress treatment was applied by adding the nutrient solution with PEG-6000 [69].

### 4.7. VIGS Experiment for Drought Stress Tolerance

The VIGS experiment used the *G. hirsutum* variety “CRI-12” to study the role of *GhTAT2* genes in drought stress tolerance and tissue-specific expression profiling via gene silencing genetic transformation. The functional analysis focused on significantly upregulated expression of the *GhTAT2* gene (*Gh_A13G1261*) in *G. hirsutum*. The candidate *GhTAT2* with a 271 bp fragment was amplified via specific forward and reverse primers. The amplified primers were subsequently cloned and inserted into the vector plasmid tobacco rattle virus (pTRV2) via *Xba*I and *Bam*HI enzymes. *A. tumefaciens* “GV3101” served as the recombinant vector carrier via the freeze-thaw method, with TRV2:*GhTAT2* as the silenced gene, TRV2:*CLA1* as the positive control and TRV2:00 as the negative control [70]. The cotton seedlings’ cotyledons underwent agroinfiltration when the cotyledons were fully expanded and before the first true leaf had just emerged. After infiltration, the plants were kept in darkness for 24 h before being transferred to a growth chamber at 25 °C with a 16/8 h light/dark cycle. Two weeks post-infection, plants transformed with the positive control gene *CLA1* presented an albino phenotype in their newly emerged leaves. 15% PEG-6000 was used to induce drought stress at the three-leaf stage, and leaf and root samples were collected at 0, 24 and 48 h in liquid nitrogen and stored at −80 °C for expression analysis in three replications from before to after treatment [71].

### 4.8. Statistical and Graphic Analysis

The data analysis was performed using one-way analysis of variance (ANOVA) at 5% probability. The significant differences between treatment means were estimated through *t*-test at the 5% and 1% (*p* ≤ 0.05 and *p* ≤ 0.01) confidence levels. GraphPad Prism (version 8.4.3) was used to display significant differences. The data are presented as the means ± SDs from three separate experiments, with * and ** indicating significance at *p* ≤ 0.05 and *p* ≤ 0.01, respectively [72].

## 5. Conclusions

A total of 203 aminotransferase genes were identified across 3 *Gossypium* species, with 103 in *G. hirsutum*, 47 in *G. arboreum*, and 53 in *G. raimondii,* through gene family analysis. Genome-wide identification, KEGG enrichment analysis, VIGS, and RT-qPCR profiling suggest that the *GhTAT2* gene cluster plays a role in drought stress tolerance through its involvement in tyrosine, cysteine and methionine, and phenylalanine metabolism. Silencing of *GhTAT2* via VIGS and subsequent RT-qPCR revealed downregulation and reduced relative expression under drought stress. *GhTAT2* has significant potential for drought stress tolerance in cotton. Thus, further gene transformation and editing are required to enhance our understanding of their roles in drought stress tolerance and metabolic mechanisms at the genetic and molecular levels.

## Data Availability

Data are contained within this article and Supplementary Materials.

## Supplementary Materials

The following supporting information can be downloaded at: https://www.mdpi.com/article/10.3390/ijms26031355/s1.

## Abbreviations

CLA	Cloroplastos alterados
CottonFGD	Cotton functional genomics database
TAT	Tyrosine aminotransferases
TRV	Tobacco rattle virus
VIGS	Virus-induced gene silencing
