# Supplementary material for: Insights into the Role of GhTAT2 Genes in Tyrosine Metabolism and Drought Stress Tolerance in Cotton
Source: Int J Mol Sci. 2025 Feb 5;26(3):1355. doi: 10.3390/ijms26031355 (PMC11818400; doi:10.3390/ijms26031355)
Supplement: Supplementary file 1 [file ijms-26-01355-s001.zip › Supplementary Materials Legends.pdf]

Supplementary Materials legends

Supplementary File S1: Physiochemical characteristics of aminotransferase genes in the A, *G. hirsutum* GhAt subgenome; B, *G. hirsutum* GhDt subgenome; C, *G. arboreum*; D, *G. raimondii*.

Supplementary Figure S1: Phylogenetics, and gene structure of the aminotransferase genes in cotton species (A), the *G. hirsutum* GhAt subgenome (B), the *G. hirsutum* GhDt subgenome (C), *G. arboreum* and (D), *G. raimondii*. The phylogenetic tree was created with MEGA 7 via the neighbor–joining method and 1000 bootstrap replicates. The exon–intron structures of the aminotransferase genes reflect their evolutionary relationships, with yellow circles representing exons and gray lines indicating introns.

Supplementary File S2: List of metabolites in aminotransferase genes and their enrichment expression after drought stress

Supplementary File S3: List of proteins of *G. hirsutum*, *G. arboreum*, *G. raimondii, A. thaliana and T. cacao*.

Supplementary File S4: List of genes and primers (forward and reverse) for RT-qPCR analysis
